# Supplementary material for: Physiological Characteristics and Transcriptomic Responses of Pinus yunnanensis Lateral Branching to Different Shading Environments
Source: Plants (Basel). 2024 Jun 7;13(12):1588. doi: 10.3390/plants13121588 (PMC11207258; doi:10.3390/plants13121588)
Supplement: Supplementary file 1 [file plants-13-01588-s001.zip › plants-3007290-supplementary.pdf]

Supporting information

Table S1. Primers used for RT-qPCR analysis

Gene name	Forward Primer Sequence (5’-3’)	Reverse Primer Sequence (5’-3’)
PsbP	CTACCCGCCGCAAACGACAAG	TCCAGCGAGCAGCGTGAGAG
PsbO	GAAGGCGGAGAGCACGAACAAG	ATCTCGTCCAGCGTGTAGGTCAG
PsaD	TTCCCCGTCCGCCGTCAG	GCCGCTGCCGACCTTGTG
PR-1	GTGCAGTGGTGGGTTAACGAGAAG	CAGCCGAGCCTCTTGGAATCTTTC
ATPase-b	GGACGCCATCTGGTTCAAGCC	ACCGCATCTGCCTCCTCCTG
AHP	TTTGAGGACTCGCTGAAGACACTG	CTTGAGCTGCTACCCTTGAAGTGG
TUBA1(Actin)	AGTCTTTTCTCGGATTGACCAC	GAGCCTCAGAGAATTCACCCT

Table S2 Sample sequencing data evaluation statistics

Sample	Raw Reads	Clean Reads	Clean Data (G)	Error Rate (%)	Q20 (%)	Q30 (%)	GC content (%)
CK_1	40448736	36765792	5.51	0	97.68	93.89	46.99
CK_2	39193410	35340862	5.30	0	97.69	93.92	46.85
CK_3	46837086	42375852	6.36	0	97.80	94.10	46.60
L1_1	39198284	35398458	5.31	0	97.69	93.93	46.16
L1_2	46639952	42218514	6.33	0	97.73	93.99	46.51
L1_3	38966630	35378956	5.31	0	97.63	93.81	46.42
L2_1	46366808	41884884	6.28	0	97.79	94.13	46.06
L2_2	45577896	41116736	6.17	0	97.62	93.74	46.28
L2_3	44024740	40052624	6.01	0	97.54	93.62	46.40
L3_1	45025894	32923696	4.94	0	97.96	94.57	46.03
L3_2	38826938	34905288	5.24	0	97.71	93.95	46.14
L3_3	40755182	36930328	5.54	0	97.55	93.59	46.23

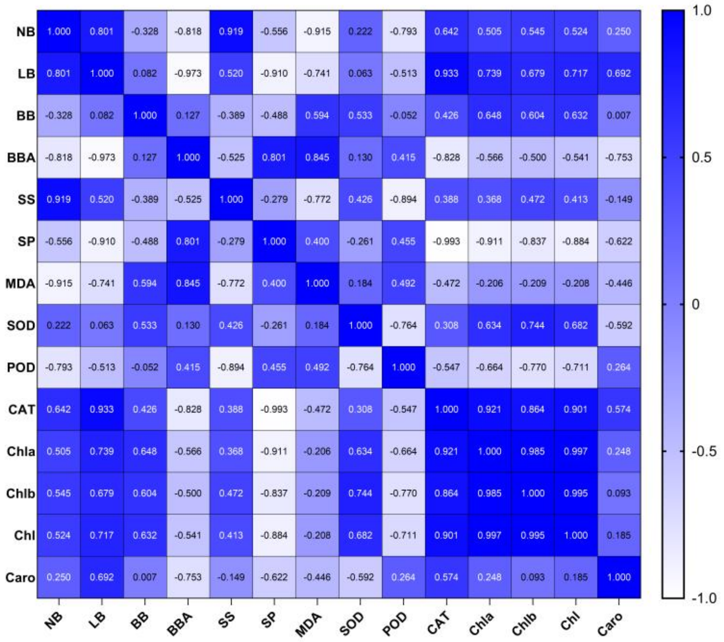

Figure S1. Pearson correlation analysis of the branching ability, photosynthetic characteristics, and physiological characteristics of *P. yunnanensis* under different shading conditions. NB: number of branches, LB: length of branches, BB: biomass of branch, BBA: branch biomass allocation, SS: soluble sugar, SP: soluble protein, MDA: malondialdehyde, SOD: superoxide dismutase, POD: peroxidase, CAT: catalase, Chla: chlorophyll a, Chlb: chlorophyll b, Chl: total chlorophyll, Caro: Carotenoid.

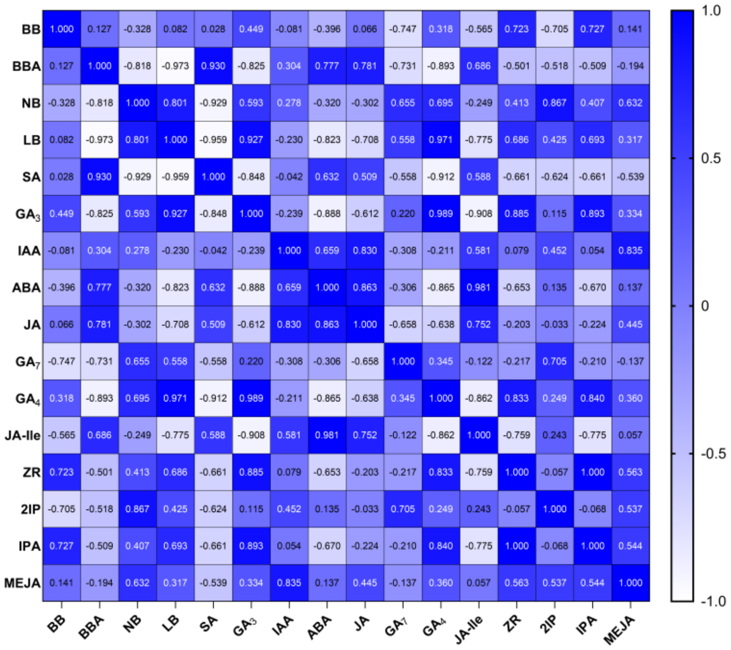

Figure S2. Pearson correlation analysis of the branching ability of *P. yunnanensis* and endogenous hormones under different shading conditions. NB: number of branches, LB: length of branches, BB: biomass of branch, BBA: branch biomass allocation, SA: salicylic acid, GA_3: gibberellin A3, IAA: auxin, ABA: abscisic acid, JA: jasmonic acid, GA_7: gibberellin A7, GA_4: gibberellin A4, JA-Ile: jasmonic acid-Isoleucine, ZR: trans-Zeatin-riboside, 2IP: isoamyl alkenyl adenine, IPA: isopentenyl-adenine, MEJA: methyl jasmonate.

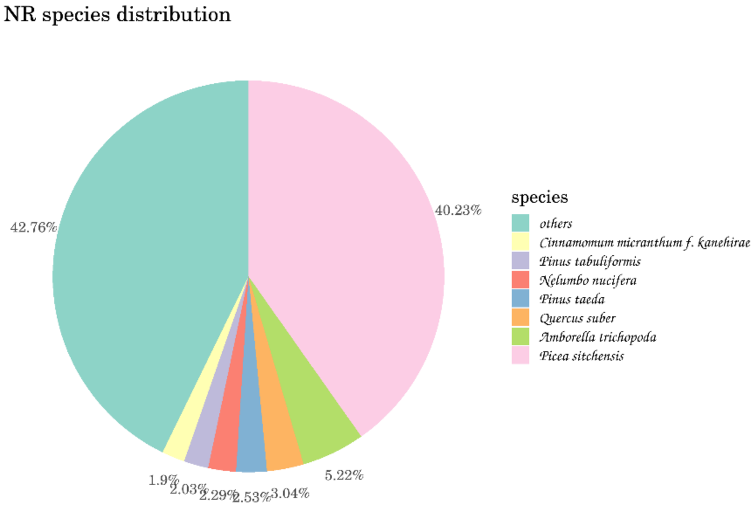

Figure S3. Species distribution of the top BLAST hits for each unigene of *P. yunnanensis* under different shading in Nr.
